# Presence of Recombinant Bat Coronavirus GCCDC1 in Cambodian Bats

**DOI:** 10.3390/v14020176

**Published:** 2022-01-18

**Authors:** Feng Zhu, Veasna Duong, Xiao Fang Lim, Vibol Hul, Tanu Chawla, Lucy Keatts, Tracey Goldstein, Alexandre Hassanin, Vuong Tan Tu, Philippe Buchy, October M. Sessions, Lin-Fa Wang, Philippe Dussart, Danielle E. Anderson

**Affiliations:** 1 Programme in Emerging Infectious Diseases, Duke-NUS Medical School, Singapore 169857, Singapore; feng.zhu@duke-nus.edu.sg (F.Z.); lxiaofan@dso.org.sg (X.F.L.); tanu.chawla@duke-nus.edu.sg (T.C.); october.sessions@nus.edu.sg (O.M.S.); 2 Virology Unit, Institut Pasteur du Cambodge, Pasteur Network, Phnom Penh 120210, Cambodia; dveasna@pasteur-kh.org (V.D.); hvibol@pasteur-kh.org (V.H.); philippe.x.buchy@gsk.com (P.B.); 3 Unité des Virus Émergents, (UVÉ: Aix-Marseille Univ-IRD 190-INSERM 1207), 13005 Marseille, France; 4 Wildlife Conservation Society, Health Program, Bronx, NY 10460, USA; lkeattsconmed@gmail.com; 5 One Health Institute, School of Veterinary Medicine, University of California, Davis, CA 95616, USA; tgoldstein@ucdavis.edu; 6 Institut de Systématique, Évolution, Biodiversité, Sorbonne Université, MNHN, CNRS, EPHE, UA, 75005 Paris, France; alexandre.hassanin@mnhn.fr; 7 Institute of Ecology and Biological Resources, Vietnam Academy of Science and Technology, No. 18, Hoang Quoc Viet Road, Cau Giay District, Hanoi 10072, Vietnam; vttu@iebr.ac.vn; 8 Peter Doherty Institute for Infection and Immunity, Department of Microbiology and Immunology, University of Melbourne, Melbourne 3000, Australia

**Keywords:** bats, coronavirus, GCCDC1, zoonosis, recombination, co-infection, cross-species transmission

## Abstract

Bats have been recognized as an exceptional viral reservoir, especially for coronaviruses. At least three bat zoonotic coronaviruses (SARS-CoV, MERS-CoV and SARS-CoV-2) have been shown to cause severe diseases in humans and it is expected more will emerge. One of the major features of CoVs is that they are all highly prone to recombination. An extreme example is the insertion of the P10 gene from reoviruses in the bat CoV GCCDC1, first discovered in *Rousettus leschenaultii* bats in China. Here, we report the detection of GCCDC1 in four different bat species (*Eonycteris spelaea*, *Cynopterus sphinx*, *Rhinolophus shameli* and *Rousettus* sp.) in Cambodia. This finding demonstrates a much broader geographic and bat species range for this virus and indicates common cross-species transmission. Interestingly, one of the bat samples showed a co-infection with an Alpha CoV most closely related to RsYN14, a virus recently discovered in the same genus (*Rhinolophus*) of bat in Yunnan, China, 2020. Taken together, our latest findings highlight the need to conduct active surveillance in bats to assess the risk of emerging CoVs, especially in Southeast Asia.

## 1. Introduction

Coronaviruses (CoV) have been known to infect humans for decades, but the severity of disease attributed to this group of viruses was only first realized when severe acute respiratory syndrome coronavirus (SARS-CoV) emerged in 2002 [1,2]. Bats were found to be the reservoir host of SARS-CoV, with zoonotic transmission to humans occurring through palm civets, an intermediate host [3]. In 2012, a decade after the SARS-CoV outbreak, Middle East respiratory syndrome coronavirus (MERS-CoV) emerged and this time the intermediate host was dromedary camels [4]. The third and undoubtedly most devastating CoV outbreak began in 2019, when SARS coronavirus 2 (SARS-CoV-2) emerged in Wuhan, China [5,6]. SARS-CoV-2 is the causative agent of the COVID-19 pandemic, and there is a global race to end the outbreak that, at the end of 2021, has led to over 286.8 million infections and 5.4 million deaths [7].

CoVs are single-stranded, positive-sense, enveloped RNA viruses, contain the largest ssRNA genome (>30 kb) of all known RNA viruses, and belong to the family Coronaviridae. CoVs are taxonomically grouped into four genera: *Alphacoronavirus*, *Betacoronavirus*, *Gammacoronavirus*, and *Deltacoronavirus*. Alpha and betacoronaviruses are known to infect humans and of the species currently classified in these genera, 54% are bat viruses [8].

It is well documented that replication of large DNA genomes is less error prone due to proofreading capacity of the DNA polymerase. In contrast to DNA polymerases, RNA dependent RNA polymerases (RdRp) lack replication fidelity [9]. CoVs are no exception and are not only prone to mutation during viral replication, but recombination. Recombination primarily occurs in the spike region [10]. Virus diversity and evolution increase the risk of zoonotic transmission as well as impacting both diagnostics and treatment.

One extreme example of a recombination event is the cross-family recombination of a Rousettus bat coronavirus (most related to HKU9) with a non-enveloped dsRNA Pteropine orthoreovirus creating a new CoV, named Rousettus bat coronavirus GCCDC1 (RoBat-CoV-GCCDC1), which incorporated a part of the reovirus p10 gene in between N and NS7a. GCCDC1 was first discovered in bats in China [11] in 2016 and then in Singapore bats in 2020 [12]. Genes p10 and NS7c are specifically present in GCCDC1 strains from China and Singapore but not in the HKU9 strain from Hong Kong.

In this study, we performed a metagenomic study to analyze bat samples from different times and geographic locations in Cambodia to investigate the presence of RoBat-CoV-GCCDC1.

## 2. Materials and Methods

### 2.1. Sample Collection

The bat sampling was performed in 2010 by the Muséum national d’Histoire naturelle (MNHN; Paris, France) and the Institut Pasteur du Cambodge (IPC) in cooperation with Cambodian Government partners. The MNHN was mandated by UNESCO and the National Authority of Preah Vihear to conduct a mammal survey in northern Cambodia. Bats were captured using mist nets and harp traps in three provinces, Preah Vihear, Steung Treng and Ratanakiri, to compare bat diversity on the two sides of the Mekong River. Selected bats captured were humanely euthanized in full compliance with local ethical and legal guidelines. Bat species were identified to genus or species level following morphological criteria and DNA barcoding.

Oral and rectal swabs were collected and stored in viral transport medium solution (VTM; containing tryptose phosphate Broth 2.95%, 145 mM NaCl, 5% gelatin, 54 mM Amphotericin B, 106 U/L penicillin-streptomycin, 80 mg/L gentamycin [Sigma-Aldrich]). All specimens were immediately transferred into liquid nitrogen containers before being transported to the Institut Pasteur du Cambodge laboratory where they were stored at −80 °C prior to testing.

Virus isolation was attempted by passaging samples on Vero-E6 (African green monkey kidney), BHK (baby hamster kidney) and LLC-MK2 (monkey kidney) and bat primary cell lines.

### 2.2. RNA Extraction

Following the manufacturer’s instructions, 750 μL TRIzol reagent (Invitrogen, Grand Island, NY, USA) was added to 250 μL of sample, followed by an addition of 200 μL chloroform and shaking for 15 s. The sample was centrifuged for 15 min at 12,000 rpm at 4 °C. The clear upper aqueous layer containing RNA, was transferred to a new 1.5 mL tube, and 0.5 mL of isopropanol per mL of initial TRIzol was added. Gentle mixing by inverting 5 times was performed before incubating for 10 min at room temperature. The sample was centrifuged for 15 min at 12,000 rpm at 4 °C. The supernatant was discarded, and the remaining pellet was resuspended in 1000 μL of 80% ethanol. The sample was again centrifuged for 5 min at 10,000 rpm at 4°C. The ethanol was removed, RNA pellet was dried and dissolved in 100% ethanol and stored at −80 °C until shipment on dry ice to Duke-NUS Medical School for sequencing.

### 2.3. Preparation of Illumina DNA Libraries from RNA

Illumina libraries were generated from total RNA with NEBNext Ultra Directional RNA Library Prep Kit for Illumina (New England Biolabs, Ipswich, MA, USA) and NEBNext Multiplex Oligos for Illumina (New England Biolabs) according to the manufacturer’s instructions with minor modifications. Briefly, 5 µL of total RNA was added to first-strand synthesis buffer and random primers followed by incubation at 94 °C for 5 min to generate RNA fragments larger than 500 nucleotides (nt). The double stranded cDNA was purified using Mag-Bind RxnPure Plus beads (Omega Bio-Tek, Norcross, GA, USA) and eluted in 60 µL nuclease-free water after first-strand and second strand cDNA synthesis. A library size between 400–600 nt was size-selected with Mag-Bind RxnPure Plus beads (Omega Bio-Tek) in a two-step selection by adding 35 µL and subsequently 15 µL of beads to the reaction. The library was eluted in 20 µL of nuclease-free water and amplified by PCR (20 cycles). Libraries were purified using the MinElute PCR Purification kit (Qiagen, Germantown, MD, USA) and eluted in 25 µL nuclease-free H_2_O. Libraries were visualized on a 1.5% agarose gel and quantified in a Bioanalyzer High Sensitivity DNA Assay (Agilent, Santa Clara, CA, USA).

### 2.4. Virus Enrichment

Targeted CoV genome enrichment was performed using a custom designed library [13] of biotinylated, 120-mer xGen Lockdown Probes (Integrated DNA Technologies, Singapore). Prior to capture of viral sequences, 1 μL of xGen Universal Blocker- TS Mix (Integrated DNA Technologies), matched according to the library index, were added to 20 μL of library DNA. A total of 0.5 μL of 5 μg Cot-1 DNA (Invitrogen) was added to block binding of probes to non-viral regions of library fragments. Blocked libraries were ethanol precipitated and resuspended in 2.5 μL of nuclease-free H_2_O, 3 μL Nimblegen hybridization solution and 7.5 μL Nimblegen 2× hybridization buffer (Roche, Mannheim, Germany) following a 10 min incubation at room temperature. The resuspended libraries were denatured at 95 °C for 10 min and cooled on ice. A total of 3 pmol of probe mix was added and hybridized to the libraries for 4 h at 65 °C. To capture virus-specific library fragments, 100 μL of magnetic M-270 streptavidin Dynabeads (Life Technologies, Waltham, MA, US) were added to the hybridization reaction and the mix was incubated for a further 45 min at 65 °C, with shaking at 2000 rpm on a ThermoMixer C (Eppendorf, Hamburg, Germany) shaker. Streptavidin beads were washed to remove unbound DNA using the SeqCap EZ hybridization and wash kit (Roche) according to the manufacturer’s instructions. A post-capture PCR amplification with P1 and P2 primers (Illumina, San Diego, CA, USA) was performed using the following conditions: initial denaturation at 95 °C for 2 min; 20 cycles of denaturation for 20 s at 95 °C, annealing for 20 s at 65 °C and extension for 15 s at 72 °C; and a final extension step for 3 min at 72 °C. The enriched library was purified using Agencourt AMPure XP beads (Beckman Coulter Genomics, Indianapolis, IN, USA) and eluted in 10 μL nuclease-free H_2_O, visualized on a 1.5% agarose gel and quantified in a Bioanalyzer High Sensitivity DNA Assay (Agilent).

### 2.5. Data Analysis

Raw reads were first imported into Geneious Prime (version 2022.0.1) for downstream analysis and trimmed of adapters with BBDuk (version 38.84). Bat host species was validated by mapping the reads to whole mitochondrial genome or specific mitochondrial genes such as CYTB or COX (depending on sequence availability) by minimap2 and identifying the best hit with highest identity and coverage. De novo assembly was conducted with clean reads by SPAdes (version 3.13.0, http://cab.spbu.ru/software/spades/, accessed on 30 December 2021) in Metagenome mode. The longest contig for each sample was then blasted against GCCDC1 reference strain 346 (NC_030886) and other GCCDC1 strains in China and Singapore to evaluate the completeness of the genome. The name RK074 was assigned to the best contig (30,161 nt) for GCCDC1. Each sample was then individually mapped to the reference RK074 genome using Geneious assembler. Annotation of RK074 was performed by comparing and transferring the annotation of China and Singapore GCCDC1 after nucleotide sequence alignment conducted by MAFFT in Geneious Prime software. Individual gene alignment was generated by Geneious alignment and used to plot the phylogeny tree by the maximum-likelihood method with the general-time-reversible (GTR) model and 1000 bootstrap replicates in PHYML 3.0 software. Phylogeny trees of different genes were exported into R, integrated with sample information and plotted by the R package “ggtree” (version 2.2.4). SNP analysis was performed in Geneious Prime and fraction of specific SNPs in different countries or host bat genus was calculated and plotted by the R package “ggseqlogo” (version 0.1). Four different bat species distribution data across the world were derived from spatial data on terrestrial mammals from the International Union for the Conservation of Nature (IUCN 2021. The IUCN Red List of Threatened Species. January 2019 [version 6.2]. https://www.iucnredlist.org; Downloaded on 18 May 2021) and exported into R (version 4.0.2). For the 4 specific species, the data were extracted with R package “tidyverse” (version 1.3.0). World map data were retrieved and cropped using the R package “rnaturalearth” (version 0.1.0) and “sf” (version 0.9–6). Distribution data were plotted on the cropped world map using r package “ggplot2” (3.3.2). Written permission from IUCN Red List was obtained for the publication of spatial data used in this study.

## 3. Results

### 3.1. Sample Collection, Virus Isolation, RNA Extraction and NGS Analysis

**Figure 1:**
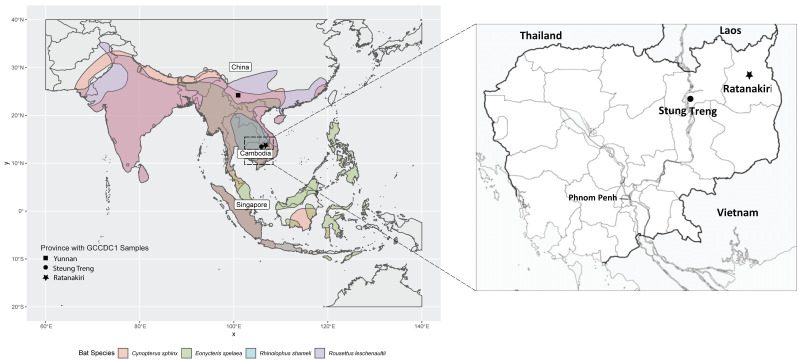
Sample location of GCCDC1 with host bat distribution map in East and Southeast Asia. Spatial distribution of four bat species were retrieved from IUCN Red List of Threatened Species version 1.18 https://www.iucnredlist.org. Downloaded on 18 May 2021. The map was further zoomed to show the two provinces as GCCDC1 sample locations in Cambodia.

A total of 10 healthy bats from four different genera were captured in the province of Steung Treng and Ratanakiri, North Cambodia during December 2010 (Table 1, Figure 1, Table S1). Oral and rectal swabs were collected and subjected to virus isolation and RNA extraction. Virus isolation was attempted on Vero-E6 (African green monkey kidney), BHK (baby hamster kidney) and LLC-MK2 (monkey kidney) and bat primary cell lines. However, the virus isolation was not successful. RNA collected from swabs was sequenced by NGS and targeted CoV genome enrichment as previously described [13]. After mapping the de novo assembly of each individual sample against known beta coronaviruses, 100% (10 of 10) returned GCCDC1 as the closest hits. Several rounds of mapping and refining of the contigs of GCCDC1 were carried out and eight of ten near-complete genomes (<10% for gaps) were assembled with two partial genomes (RK072 and RK022) covering 55% and 82.7%, respectively (Table 2). The best quality genome with 100% coverage was named RK074. Each genome was compared against the GCCDC1 strains found in China and Singapore. All genomes from Cambodian bats shared highest sequence identities with GCCDC1 346 among GCCDC1 356 from China and GCCDC1 from Singapore. It is interesting to note that GCCDC1 was found in four different bat species in Cambodia, covering the species diversity previously found in the Chinese and Singaporean studies [11,12], making Cambodia the overlapping “center” region for GCCDC1 bat hosts.

**Figure 2:**
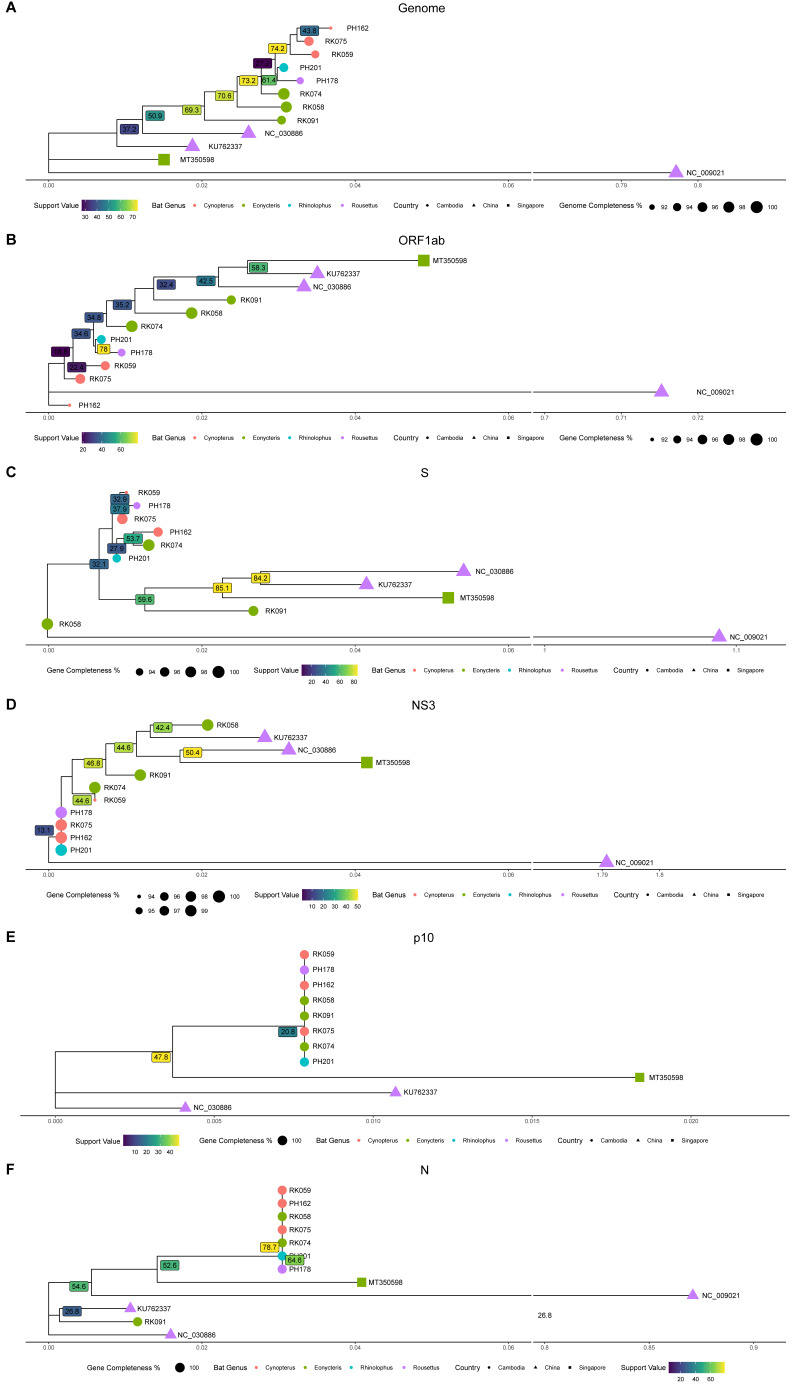
Phylogenetic analysis of GCCDC1. Phylogenetic trees based on the (**A**) whole genome, (**B**) RdRp, (**C**) spike, (**D**) NS3, (**E**) p10, and (**F**) N gene sequences. Beta coronavirus HKU9 was used as the outgroup. Trees were generated using PhyML with general-time-reversible (GTR) substitution model and 1000 bootstrap replicates. Numbers above or below the branches are percentage bootstrap values for the associated nodes. The x axis represents the number of substitutions per site.

Depending on the genomic regions used for phylogenetic analysis, the trees displayed slightly different topologies (Figure 2). However, it is clear that the CoV strains detected in Cambodian bats, albeit from different bat species, are clustered more closely than those detected in China or Singapore.

### 3.2. Bat Species-Specific Features

**Figure 3:**
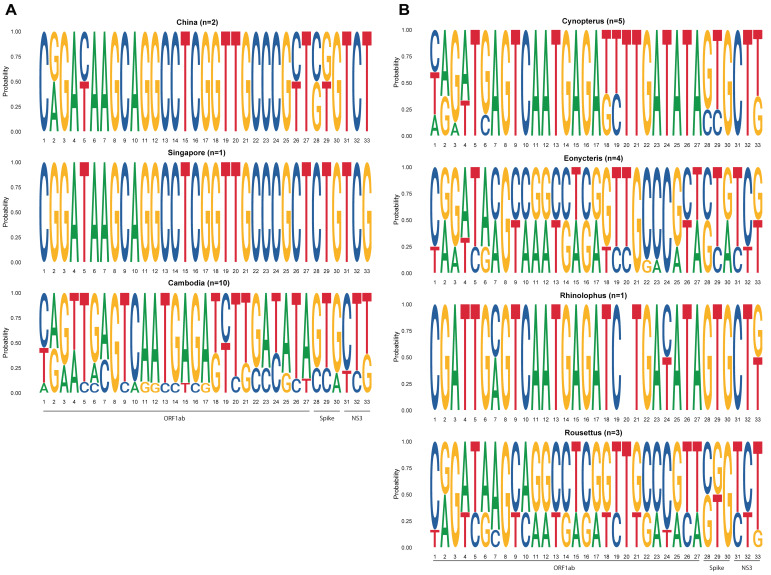
Analysis on non-synonymous mutations in GCCDC1 in countries and host bat genus. A total of 33 sites were selected to calculate the frequencies of non-synonymous mutations in (**A**) different countries and (**B**) host bat genus. Locations of the sites and effect on protein sequence can be found on Sup Table 1. Due to mixture of reads in some samples, non-ATGC nucleotides were transferred into A or T or G or C in equal fractions.

To investigate the bat species-specific features of GCCDC1, we performed whole-genome SNP analysis for all previous and current GCCDC1 strains in this study. A total of 33 sites in ORF1ab, Spike and NS3 genes were analyzed to compare the frequencies of non-synonymous mutations between different countries and host bat genus (Figure 3, Table S2). These 33 sites were chosen as they were non-synonymous substitutions occurring more than once in the examined sequences. There were no unique SNPs in GCCDC1 Chinese or Singaporean strains, while Cambodian strains had many. Both geographic region and host bat genus favoured some SNPs over others. For example, at Position 4, while the proportion of a T was quite similar in *Cynopterus, Eonycteris* and *Rousettus* host bats, there were none in China or Singapore strains. At Position 7, C was a hallmark for Cambodian strains, but not in the *Cynopterus* host. The *Cynopterus* and *Rhinolophus* host carried more unique SNPs than the *Eonycteris* and *Rousettus* strains. 

### 3.3. RsYN14-Like AlphaCoV in PH201

**Figure 4:**
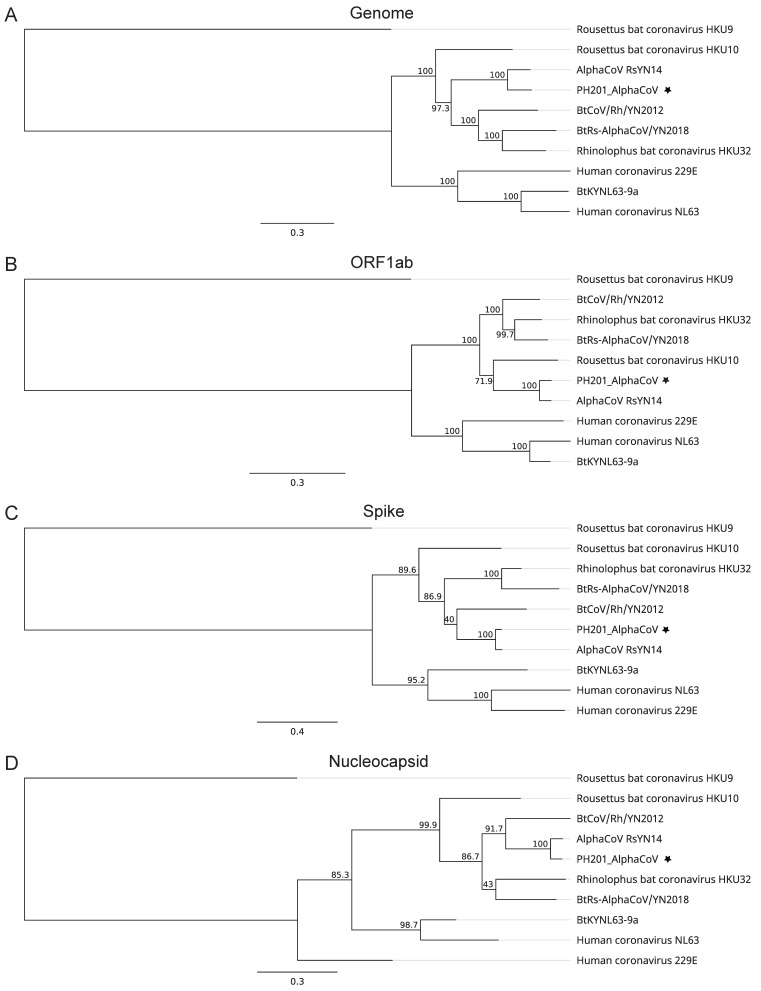
Phylogenetic analysis of PH201 AlphaCoV. Phylogenic trees were based on the (**A**) whole genome or (**B**) RdRp, (**C**) spike and (**D**) N gene sequences. Beta coronavirus HKU9 was used as the outgroup. Trees were generated using PhyML with general-time-reversible (GTR) substitution model and 1000 bootstrap replicates. Numbers above or below the branches are percentage bootstrap values for the associated nodes. The x bar represents the number of substitutions per site. The PH201 AlphaCoV is marked with a star symbol.

For bat sample PH201, an alphaCoV was found to be present in addition to GCCDC1, indicating a co-infection of two CoVs in two different genera. During sequence analysis and after removing the reads mapped to GCCDC1, the remaining reads could also be assembled into a near full genome (96.7% coverage) of a novel alphaCoV. The closest virus is RsYN14 which was discovered in the same genus of bat in Yunnan, China, 2020 [14]. The nucleotide sequence identity between the full genome PH201_AlphaCoV detected in Cambodia and RsYN14 is 83.2% (Table 3). Closer investigation at the gene level revealed that PH201_AlphaCoV shares an average of 89.5% protein sequence identity with RsYN14 for all genes, ranging from ORF4a at 76.3% to M at 98.4%. Phylogenetic analysis based on the whole genome or individual genes (Figure 4) inferred the closeness of RsYN14 and PH201_AlphaCoV, with the second-closest virus being either *Rousettus* coronavirus HKU10 or *Rhinolophus* coronavirus Bt-CoV/Rh/YN2012, depending on the genes.

## 4. Discussion

Better understanding of potentially zoonotic viruses, such as bat CoVs, is highly important for risk assessment of potential spillover events which may lead to human-to-human transmission. Due to the high frequency of genome-level recombination, CoV evolution is dynamic and evolving virus variants poses a risk to public health.

Here we report the presence of multiple strains of the cross-family recombinant RoBat-CoV GCCDC1 in Cambodian bats. The authenticity of GCCDC1 found in Cambodia is confirmed by the close genetic relationship with those found in China and Singapore, as well as the presence and sequence conservation of the reovirus p10 gene located between N and NS7a.

This study highlights several significant observations. First, it indicates a wider geographic distribution of GCCDC1 in Asia than previously known. It will be interesting to investigate whether GCCDC1 is present in bats outside of Asia. Second, our data suggest cross-bat species infection by GCCDC1 can happen quite frequently as bats from all four genera sampled in this study were positive for GCCDC1. Third, SNP analysis indicated some bat species-specific associations could possibly be confounded by bat spatial distribution. Fourth, the detection of GCCDC1 in Cambodia in all bat species previously found in China or Singapore underscore not only the “central” location of Cambodia, but also the richness of both bat and virus diversity in Cambodia. Finally, the detection of a novel alphaCoV co-infected with GCCDC1 further highlights the propensity for co-evolution or spill-over of CoVs among different bat species. A recently discovered SARS-CoV-2 related bat coronavirus in Cambodia [15] was found in the same area, in the same host species (*Rhinolophus shameli*) and at the same time of sampling, suggesting the urgency of more detailed investigation and monitoring of co-evolution.

In conclusion, this study demonstrates the wide geographic location of an evolutionally dynamic recombinant bat CoV. Enhanced surveillance in a broad geographical area, in both bats and humans is required to monitor the evolution of this group of viruses and to assess potential zoonotic transmission into human populations.

## Figures and Tables

**Table 1:** Bat sampling.

Animal ID	Country	Province	Village	Family	Genus	Species	Type of Sample	Date Sample Taken
PH162	Cambodia	Steung Treng	Chhab Village	Pteropodidae	*Cynopterus*	sp.	Rectal swab	5–6-December-2010
PH178	Cambodia	Steung Treng	Chhab Village	Pteropodidae	*Rousettus*	sp.	Oral swab	6–7-December-2010
PH201	Cambodia	Steung Treng	Chhab Village	Rhinolophidae	*Rhinolophus*	*shameli*	Rectal swab	6–7-December-2010
RK022	Cambodia	Ratanakiri	Pouy Village	Pteropodidae	*Cynopterus*	sp.	Rectal swab	14–15-December-2010
RK058	Cambodia	Ratanakiri	Ta Kouy Village	Pteropodidae	*Eonycteris*	*spelaea*	Rectal swab	15–16-December-2010
RK059	Cambodia	Ratanakiri	Ta Kouy Village	Pteropodidae	*Cynopterus*	*sphinx*	Rectal swab	15–16-December-2010
RK072	Cambodia	Ratanakiri	Veung Seing Village	Pteropodidae	*Cynopterus*	sp.	Rectal swab	17–18-December-2010
RK074	Cambodia	Ratanakiri	Veung Seing Village	Pteropodidae	*Eonycteris*	*spelaea*	Rectal swab	17–18-December-2010
RK075	Cambodia	Ratanakiri	Pan Porng Village	Pteropodidae	*Cynopterus*	sp.	Rectal swab	18–19-December-2010
RK091	Cambodia	Ratanakiri	Pan Porng Village	Pteropodidae	*Eonycteris*	*spelaea*	Rectal swab	18–19-December-2010

**Table 2:** Genomic features for each of the ten GCCDC1 strains characterization by NGS.

			MT350598 (GCCDC1 Singapore)	NC_030886 (GCCDC1 356)	KU762337 (GCCDC1 346)	ORF1ab			S			NS3			E			M			N			p10			NS7a			NS7b			NS7c		
ID	Length	X^#^	Genome	Genome	Genome	nt	aa	x^#^	nt	aa	x^#^	nt	aa	x^#^	nt	aa	x^#^	nt	aa	x^#^	nt	aa	x^#^	nt	aa	x^#^	nt	aa	x^#^	nt	aa	x^#^	nt	aa	x^#^
PH162	30101	90.9	90.49	92	93.81	90.9	90.5	92	93.8	94.8	97	97.5	97	100	27.9	8.31	3.9	86.7	84.8	87	96.5	97.7	100	98.2	98.9	100	97.3	100	100	97.9	98.5	100	66.3	57.1	56
PH178	30101	93	92.84	94.4	92.07	93	92.8	94	92.1	93.1	94	97.5	97.4	100	28.9	8.31	5.2	97.4	97.7	100	96.5	97.7	100	98.2	98.9	100	97.3	100	100	97.9	98.5	100	73.6	60.1	69
PH201	30134	94.2	94.8	95.6	93.53	94.2	94.8	96	93.5	95.2	96	97.6	97.4	100	69.2	47.3	75	97.3	97.7	100	96.5	97.7	100	98.2	98.9	100	97.3	100	100	97.9	98.5	100	97.3	96.8	98
RK022	30113	82.8	77.79	81.8	83.93	82.8	77.8	82	83.9	81.1	83	89.4	85.7	90	27.9	8.31	3.9	82.2	78	78	96.4	97.5	100	97.5	96.8	100	97.3	100	100	97.9	98.5	100	78.1	68.9	75
RK058	30139	97.4	99.12	100	96.67	97.4	99.1	100	96.7	99.4	100	97.8	98.3	100	100	100	100	97.4	97.7	100	96.5	97.7	100	98.2	98.9	100	97.3	100	100	97.9	98.5	100	86.2	83.3	82
RK059	30101	93.8	94.31	95.9	91.01	93.8	94.3	96	91	91.7	93	92.8	91.9	94	25	4.55	0	92	90.5	91	96.5	97.7	100	98.2	98.9	100	97.3	100	100	97.9	98.5	100	97.5	96.1	98
RK072	29961	63.1	51.22	55.4	51.64	63.1	51.2	55	51.6	36.7	41	53.7	39.6	42	25	4.55	0	82.6	70.1	86	64.4	52.5	60	94.8	89.3	100	93.8	93.8	96	79.8	87.7	92	50.3	35.7	39
RK074	30145	97.6	99.43	100	96.45	97.6	99.4	100	96.5	99.2	100	97.2	97.4	100	100	100	100	97.3	97.7	100	96.5	97.7	100	98.2	98.9	100	97.3	100	100	97.9	98.5	100	99.6	100	100
RK075	30147	94.9	96.2	97.2	95.29	94.9	96.2	97	95.3	97.4	98	97.3	97	100	28.9	8.31	5.2	97.3	97.7	100	96.5	97.7	100	98.2	98.9	100	97.3	100	100	97.9	98.5	100	85.4	82.1	81
RK091	30147	94.3	94	96.1	94.04	94.3	94	96	94	95.1	98	96.1	92	100	98.3	100	100	96.4	96.8	100	98.1	99.6	100	98.2	98.9	100	97.3	100	100	99.5	98.8	100	52.3	39.8	36

#: X represents coverage, shown in percentage.

**Table 3:** Genomic characteristics of AlphaCoV in PH201 bat.

ID	Length	X^#^	MZ08395 (RsYN14)	ORF1ab			S			ORF3a			ORF4a			E			M			N			ORF8			ORF9		
				nt	aa	x^#^	nt	aa	x^#^	nt	aa	x^#^	nt	aa	x^#^	nt	aa	x^#^	nt	aa	x^#^	nt	aa	x^#^	nt	aa	x^#^	nt	aa	x^#^
PH201	28807	96.7	83.23	82.67	90.37	96.9	80.36	91.09	96.9	85.67	90.61	100	77.94(early termination)	76.29(early termination)	100	90.89	93.55	93.8	95.22	98.35	100	86.75	91.48	100	89.89	90.91	100	83.77	80.79	100

#: X represents coverage, shown in percentage.

## Data Availability

Genomic sequences reported in this study have been deposited in GenBank under the accession number OM219639 to OM219649. Raw sequence reads reported in this study have been uploaded into SRA under BioProject ID PRJNA795576.

## Supplementary Materials

The following supporting information can be downloaded at: https://www.mdpi.com/article/10.3390/v14020176/s1, Table S1: bat species identification; Table S2: selected SNP sites for analysis.
